# Characterization of Pearl Millet Root Architecture and Anatomy Reveals Three Types of Lateral Roots

**DOI:** 10.3389/fpls.2016.00829

**Published:** 2016-06-13

**Authors:** Sixtine Passot, Fatoumata Gnacko, Daniel Moukouanga, Mikaël Lucas, Soazig Guyomarc’h, Beatriz Moreno Ortega, Jonathan A. Atkinson, Marème N. Belko, Malcolm J. Bennett, Pascal Gantet, Darren M. Wells, Yann Guédon, Yves Vigouroux, Jean-Luc Verdeil, Bertrand Muller, Laurent Laplaze

**Affiliations:** ^1^UMR DIADE, Institut de Recherche pour le Développement, MontpellierFrance; ^2^UMR AGAP, Centre International de Recherche Agronomique pour le Développement–Virtual Plants, Institut National de Recherche en Informatique et en Automatique, MontpellierFrance; ^3^Laboratoire Mixte International Adaptation des Plantes et Microorganismes Associés aux Stress Environnementaux, DakarSénégal; ^4^Laboratoire Commun de Microbiologie IRD/ISRA/UCAD, DakarSénégal; ^5^UMR DIADE, Université de Montpellier, MontpellierFrance; ^6^Laboratoire d’Ecophysiologie des Plantes sous Stress Environnementaux (UMR LEPSE, INRA-Supagro), MontpellierFrance; ^7^Centre for Plant Integrative Biology, School of Biosciences, University of Nottingham, Sutton BoningtonUK; ^8^Centre d’Etude Régional pour l’Amélioration de l’Adaptation à la Sécheresse, Institut Sénégalais des Recherches Agricoles, ThièsSénégal; ^9^Plateforme PHIV, UMR AGAP, Centre International de Recherche Agricole pour le Développement, MontpellierFrance

**Keywords:** lateral root, root growth, metaxylem, root architecture, breeding

## Abstract

Pearl millet plays an important role for food security in arid regions of Africa and India. Nevertheless, it is considered an orphan crop as it lags far behind other cereals in terms of genetic improvement efforts. Breeding pearl millet varieties with improved root traits promises to deliver benefits in water and nutrient acquisition. Here, we characterize early pearl millet root system development using several different root phenotyping approaches that include rhizotrons and microCT. We report that early stage pearl millet root system development is characterized by a fast growing primary root that quickly colonizes deeper soil horizons. We also describe root anatomical studies that revealed three distinct types of lateral roots that form on both primary roots and crown roots. Finally, we detected significant variation for two root architectural traits, primary root lenght and lateral root density, in pearl millet inbred lines. This study provides the basis for subsequent genetic experiments to identify loci associated with interesting early root development traits in this important cereal.

## Introduction

In Africa, most of the recent increase in agricultural production has been due to the expansion of cultivated lands rather than an increase in yields (Bationo et al., 2007). Moreover, several climate models predict that global changes may reduce the potential productivity of cereals (Berg et al., 2013). For example, millets potential productivity is predicted to decrease by 6% in the driest cultivated regions. In order to achieve future food security in Africa, it is therefore necessary to improve crop productivity through breeding and improved agricultural practices.

Pearl millet [*Pennisetum glaucum* (L.) R. Br.] is the sixth most important cereal grain in the world (Food and Agriculture Organization of the United Nation [FAO], 2014). It accounts for 6% of the total cereal production in Africa, and 14% in West Africa alone (Food and Agriculture Organization of the United Nation [FAO], 2014). Pearl millet grain is a significant source of micronutrients such as iron and zinc with contents higher than those in other cereals (Souci et al., 2000). Both in sub-Saharan Africa and India, it potentially represents one of the cheapest food sources of these micronutrients and proteins when compared with other cereals and vegetables. In addition, pearl millet is well adapted to dry climates and is mostly grown in areas with limited agronomic potential characterized by low rainfall, in the 200–500 mm range, and marginal soils (Guigaz, 2002). These facts make millet an important food staple over much of the African continent, especially in the semi-arid areas of the Western Sahel where other crops tend to fail because of inadequate rainfall and poor soil conditions. Thus pearl millet is an important cereal in arid and semi-arid regions where it contributes to food security and is expected to have an increased importance in the future adaptation of agriculture to climate change in sub-Saharan Africa.

Despite its importance, pearl millet is considered as an orphan crop because it has received very little support from science, industry and politics while other crops such as wheat, rice, or maize were subjected to intense efforts of genetic and agronomic improvement. As a result, it lags behind sorghum and far behind the other major cereals in its genetic improvement. Its average grain yields barely reach 900 kg/ha, compared to 1500 kg/ha for sorghum (Food and Agriculture Organization of the United Nation [FAO], 2014). Moreover, production has increased by only 0.7% a year in West Africa during the last two decades, the lowest growth rate of any food crop in the region and far less than the population’s growth rate of nearly 3% per year (United Nations Statistics Division, 2016). However, its untapped genetic potential is vast and could be used to improve pearl millet tolerance to some environmental factors that are the main limitations to its growth potential. For instance, pearl millet is mostly grown in marginal soils such as sandy soils in Western Sahel where low water and nutrient (particularly phosphate) availability are major limiting factors. Moreover, root establishment in poor soil is essential to ensure efficient use of available water.

The importance of root architecture for water and nutrient acquisition has been well documented in both monocots and dicots, and could be successfully used for root trait-targeted genetic improvement. For example, targeted modifications of root architecture in pea to increase P acquisition efficiency were achieved (Lynch, 2011). Pearl millet is a monocot species displaying a fibrous root system in which different categories of roots can contribute to a various extent in root system growth, branching and tropism dynamics as well as to water transport. Importantly, substantial differences in root traits were reported for eight pearl millet varieties grown in soil in Niger (Brück et al., 2003) indicating a potential genetic diversity that could be used for breeding and selecting new varieties with improved root systems. However, the detailed structure and dynamics of pearl millet root system has not been described and very little is known about root growth and anatomy.

Here, we analyzed root architecture during the early phase of pearl millet development. Furthermore, we identified and characterized the anatomy of the different root types. Finally, we compared two root development parameters in 16 pearl millet inbred lines and show that there is a large diversity of phenotypes that could be exploited in later breeding studies.

## Materials and Methods

### Plant Material

Pearl millet [*Pennisetum glaucum* (L.) R. Br.] inbred lines (Saïdou et al., 2009) originating from Indian, West and Central African landraces were used in this study. Seeds were surface sterilized with 5% hypochlorous acid for 2 min, rinsed three times in sterile water, then immerged in 70% ethanol for 2 min, rinsed three times again and kept for 10 min in sterile water. Seeds were put in Petri dishes containing wet filter paper for 24 h in the dark at 30°C for germination. The age of the plants are given in DAG (days after germination), i.e., the number of days from the date of seed- transfer onto the filter paper for germination.

### Root Phenotyping

**FIGURE 1:**
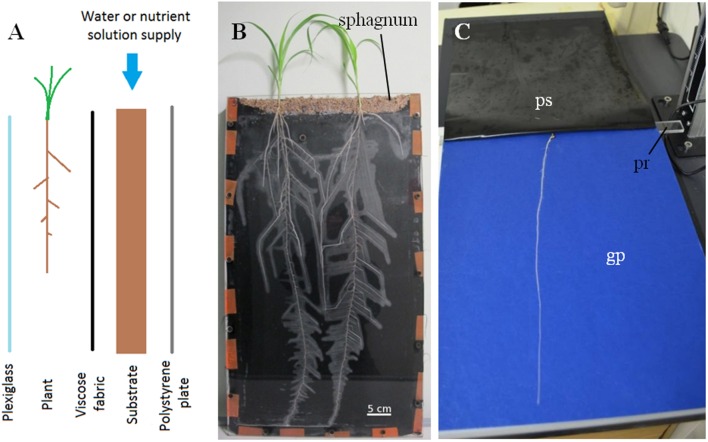
**(A)** Scheme of the rhizotron used. **(B)** A rhizotron at the end of an experiment. Scale bar: 5 cm. **(C)** One of the pouches used in the high-throughput phenotyping system. ps, plastic sheet; pr, plastic rod; gp, germination paper. These three elements are held together by foldeback clips (not visible here).

For analysis of root development, rhizotrons were built according to Neufeld et al. (1989). They were composed of a 400 mm × 700 mm × 20 mm aluminum frame, and, from rear to front, a 5 mm extruded polystyrene layer, a 20 mm layer of substrate, a cellulose acetate tissue layer (40 μm mesh) and a 5 mm plexiglass (**Figure 1A**). In this system, the root system grows in two dimensions between the fabric and the plexiglass (**Figure 1B**). The cellulose acetate was chosen because it is both non-deformable, preventing roots to grow through (this was confirmed at harvest), and allows roots to remain hydrated. The water content of the substrate was evaluated at the onset of the experiment and later maintained above stressful threshold by daily weighing the rhizotrons and watering from the top. The substrate used was composed of 30% fine clay, 25% peat fibers, 5% blond peat, and 40% frozen black peat (Klasmann–Deilmann France SARL). The average SWC (Soil Water Content) of the substrate was 56% (w:w). At 1 DAG, one germinated seedling (displaying a primary root of about 1 cm long) was transferred to the top of each rhizotron, in a layer of wet sphagnum. This layer was permanently maintained wet in order to prevent the seedlings from drying out during the early stages of growth. The plants were placed in a 1 m^2^ growth room with a 14 h photoperiod, a temperature of 28°C/24°C during days/nights and a VPD of 1.5 kPa. From the second day of growth onward, rhizotrons were scanned (Epson Expression 10000XL) every day at a fixed time at a resolution of 600 DPI. Root system outlines were then extracted using SmartRoot (Lobet et al., 2011). These outlines comprised information on all root lengths, branching position and angle for every scan.

For high-throughput root phenotyping, a paper-based system was used (**Figure 1C**) according to Atkinson et al. (2015). One DAG-old seedlings were transferred into pouches and then maintained in a growth room with a 14 h photoperiod (28°C during day and 24°C during night). Pictures of the root system were taken every 2 days for 6 days with a D5100 DSLR camera (Nikon) at a resolution of 16 M pixels. The camera was fixed on a holder to maintain the same distance between the lens and each root system. At 6 DAG, the root tip of the “fastest-growing” plants reached the bottom of the pouches. The experiment was repeated four times independently. Root traits (primary root length, lateral root density along the primary root and number of crown roots) were extracted using RootNav (Pound et al., 2013).

### Root Sections and Microscopy

One DAG-old seedlings were transferred in a hydroponic system containing quarter strength Hoagland medium (Hoagland and Arnon, 1950) or put on the top of seed germination paper (Anchor Paper Company, USA) rolled on itself with the base immerged in distilled water (Hetz et al., 1996). The plants were kept in a growth chamber (12 h photoperiod, a temperature of 27°C and an hygrometry of 60%) for 10–20 days. For sections of fresh material, 1-cm long samples were collected at the root apex and every 5 cm along the root and were embedded in agarose blocks (3% v/v in water) before sectioning, as described in Lartaud et al. (2014). The sampling positions were recorded. Transverse root sections (thickness 60 μm) were obtained using a HM 650 V vibratome (Microm) and observed directly under the epifluorescence microscope. Some section were stained with Safranin and Alcian blue (FASGA, Tolivia and Tolivia, 1987).

For thin sections, samples were fixed and dehydrated as described by Scheres et al. (1994). Samples were then embedded in Technovit 7100 resin (Heraeus Kulzer) according to the manufacturer’s instructions. Thin longitudinal sections (5 μm) were produced with a HM355S microtome (Microm). Sections were stained for 15 min in aqueous 0.01% toluidine blue (pH = 6,8) solution and mounted in Clearium Mountant (Surgipath). Sections were visualized using a Leitz DMRB epifluorescence microscope [objectives used: 10×, numerical aperture (NA) = 0,3; 20×, NA = 0,5; 40×, NA = 0,75]. Pictures were taken using a Retiga SRV FAST 1394 camera (QImaging) and the QCapture Pro7 software (QImaging). Vessel dimensions were measured using ImageJ.

### X-Ray Microcomputed Tomography

Plants were transferred to pots (50 mm diameter and 120 mm height) containing “Newport Series Loamy Sand” soil [sand 83.2%, silt 4.7%, and clay 12.1%; organic matter 2.93%; pH = 7.13; Nitrate = 5.48 mg.L^-1^; Phosphorus = Defra index of 3 (29.65 mg kg^-1^)] 1 DAG. Plants were maintained throughout the experiment at a soil water content of ~26% (w:w), which corresponds to 75% of field capacity. The SWC was monitored daily by weighing the pots. Plants were scanned with a v| tome| x M scanner (Phoenix/GE Systems), with a maximum energy of 240 kV, four times over an 18 days period (4, 8, 14, and 18 DAG) to image the root structure. Root systems were segmented manually from the image stacks using the VGStudio Max software (Volume Graphics GmbH).

### Statistical Analyses and Heritability Estimates

Statistical analyses were performed using R (R Development Core Team, 2008). An analysis of variance was performed to detect an effect of the line on the variability of the different root traits measured. When an effect was detected, a Tukey’s HSD (Honest Significant Difference) test was used to group lines of homogeneous means for the trait of interest.

Broad sense heritability was computed by dividing the variance associated with line with the total variance of the character (variance associated with line + environmental variance + residual variance).

Average seed weight for each line was evaluated and a Spearman’s rank correlation coefficient was computed to detect a putative correlation between seed weight and root trait.

## Results

### Early Development of Pearl Millet Root System

**FIGURE 2:**
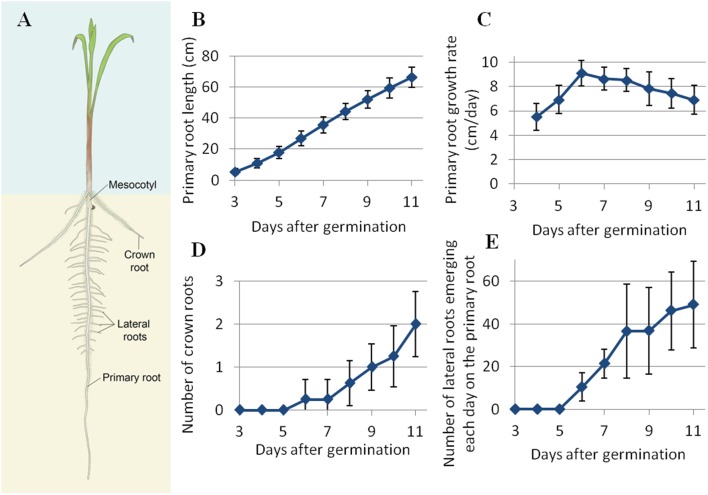
**(A)** Scheme of the various roots of a pearl millet seedling. **(B)** Daily average length of the primary root. **(C)** Daily average primary root growth rate. **(D)** Daily cumulative number of lateral roots along the primary root. **(E)** Daily cumulative number of crown roots. *N* = mean ± standard deviation.

The emergence and development of different roots in pearl millet seedling was studied in different growth conditions. Different roots observed at early stage are named according to the nomenclature presented in **Figure 2A**, based on the nomenclature used for maize root systems (Hochholdinger and Tuberosa, 2009). The first root to emerge from the seed, initially called the radicle, is then called the primary root. A small segment, called the mesocotyl, links the seed and the base of the shoot. At later stages of development, crown roots emerge from the base of the shoot. Branches that appear on the primary or crown roots are called lateral roots. The lateral roots can branch themselves, these ramifications being called secondary lateral roots.

The developmental dynamics of the root system was studied more finely on pearl millet line LCICMB1 (line 109 of the panel). In all of the plants that we analyzed in rhizotrons (*n* = 28), the early root system of pearl millet was made up of a single primary root that has emerged from the seed 12 to 24 h after seed rehydration. This primary root grew vertically at an increasing rate during the first 6 DAG, reaching a maximum of 9.1 cm day^-1^ (**Figures 2B,C**). After that date, the primary root growth rate slightly slows down, but remains *ca.* 7 cm day^-1^ at 11 DAG (**Figure 2C**). The average primary root length at 11 DAG was 66.3 cm (**Figure 2B**). Crown roots and lateral roots started to emerge, respectively, from the shoot base and on the primary root at 6 DAG. The average number of crown roots per plant is shown in **Figure 2D**. Crown roots started to emerge 6 DAG and were in average two per plant at the end of the experiment. This number is quite low and this experiment only captured the very beginning of crown root emergence period. Average crown root growth rate was 3.7 cm day^-1^. The number of lateral roots emerging each day on the primary root is shown on **Figure 2E.** Lateral roots started to emerge on the primary root 6 DAG. Their emergence rhythm increased until the end of the experiment, quickly up to 8 DAG and then slowly between 8 and 11 DAG. Lateral root density on the primary root was 4.2 roots cm^-1^. Lateral root growth rates were heterogeneous, reaching up to 3 cm day^-1^. Interestingly, crown roots and lateral roots started to appear at 6 DAG, when primary root growth rate reached its maximum, and correlates with the emergence of the third leaf.

**FIGURE 3:**
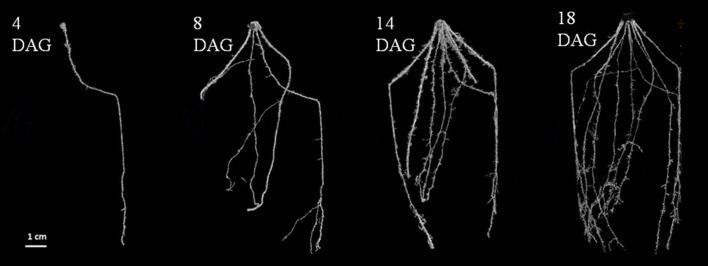
**Establishment of the architecture of a soil grown pearl millet root system using X-Ray CT: 2D projection of a 3D image of the root system architecture.** Images at 4, 8, 14, and 18 DAG (days after germination). Scale bar: 1 cm

Early root development was also analyzed in 3D in soil using micro-computed x-ray tomography (**Figure 3**). LCICMB1 plants were grown in small soil columns (5 cm diameter × 12 cm high) and scanned at 4, 8, 14, and 18 DAG. As in the rhizotrons, only primary root was visible at 4 DAG and crown and lateral roots could be detected from 8 DAG onward. This indicated that these roots emerged between 4 and 8 DAG, but the time resolution was too rough to identify a precise emergence date. However, this time interval is consistent with their emergence time observed in rhizotron, of 6 DAG. This observation therefore supports the hypothesis that rhizotrons provide a realistic assessment of root architecture development in natural conditions. The 3D images also gave us information about the organization of the different roots in space. The primary root, first to emerge, grew nearly vertically into the soil volume. On the contrary, crown roots grew at an angle of between 20° and 40° to vertical. This angle appeared conserved for the first centimeters of crown root growth, but the small diameter of the pots scanned constraining root growth to just a few centimeters after emergence, did not allow us to check whether this angle could be maintained. Crown root emergence sites were distributed regularly in space around the stem base.

Hence, early root system development in pearl millet is characterized by a fast growing primary root that quickly colonizes deeper soil horizons, while lateral and crown roots only start to emerge 6 DAG.

### Anatomy of the Different Root Types

We next analyzed the cellular organization of primary, crown and lateral roots of young pearl millet plants (LCICMB1 line) grown on germination paper or in hydroponics. Root fragments were harvested at different positions along the root and transverse sections were obtained using a vibratome. As root characteristics did not vary strongly in the zone we sampled (Supplementary Figure 1 for example of stele diameter) we considered all the samples we had to define the anatomical features of the different root types (**Table 1**).

**Table 1:** Anatomical features of the different root types in pearl millet.

Root type	Root diameter (μm)	Stele diameter (μm)	# Metaxylem vessels	Metaxylem vessel diameter (μm)	*n*
Primary root	429 ± 103^ab^	181 ± 34^b^	1	58 ± 11^a^	10
Crown root	517 ± 76^a^	229 ± 54^a^	3	56 ± 9^a^	8
LR type 1	112 ± 27^d^	32 ± 8^e^	0	NA	14
LR type 2	264 ± 22^c^	74 ± 9^d^	1	16 ± 2^b^	7
LR type 3	367 ± 66^b^	145 ± 16^c^	1	50 ± 6^a^	12

*Mean and standard deviation of all sections. Letters correspond to groups formed by Tukey’s Honest Significant Difference test (alpha = 0.05). *n*, sample size.*

**FIGURE 4:**
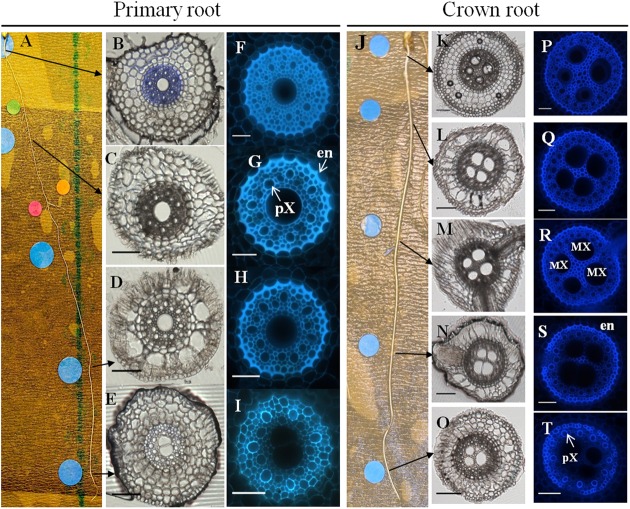
**Anatomical organization of a primary root **(B–I)** and a crown root **(K–T)**, 11 and 15 DAG, respectively.** Transverse sections were performed every 5 cm, from the root apex to the root basis. **(A)** General view of a primary root with the sampled zones marked by an arrow. **(B–E)** Transverse section of primary root observed in transmitted light (scale bar: 100 μm). **(F–I)** Transverse section of primary root focused only on the root stele, observed in epifluorescence (natural autofluorescence at 460–480 nm; scale bar: 50 μm). **(J)** General view of a crown root with the sample zones marked. **(K–O)** Transverse section of crown root observed in transmitted light (scale bar: 100 μm). **(P–T)** Transverse section of crown root focused only on the root stele, observed in epifluorescence (natural autofluorescence at 460–480 nm; scale bar: 50 μm) co, cortex; ae, aerenchyma; MX, metaxylem; pX, peripheric xylem vessel; en, endodermis.

Primary roots were characterized by a large diameter metaxylem vessel located at the center of the stele (**Figure 4**). Their ground tissue contained 3–5 layers of cortical cells. Aerenchyma differentiation was observed in mature parts of the root. Crown roots were thicker than primary roots with a significantly larger stele that contained 2–5 (three in most cases) large metaxylem vessels separated by parenchyma cells (**Figure 4**, **Table 1**). They also showed 3–5 layers of cortical cells and aerenchyma. In both cases, cell wall autofluorescence was lower in the stele close to the root tip and increased particularly in the endodermis as the root matures, presumably because of cell wall lignification and suberization accompanying casparian strip formation.

**FIGURE 5:**
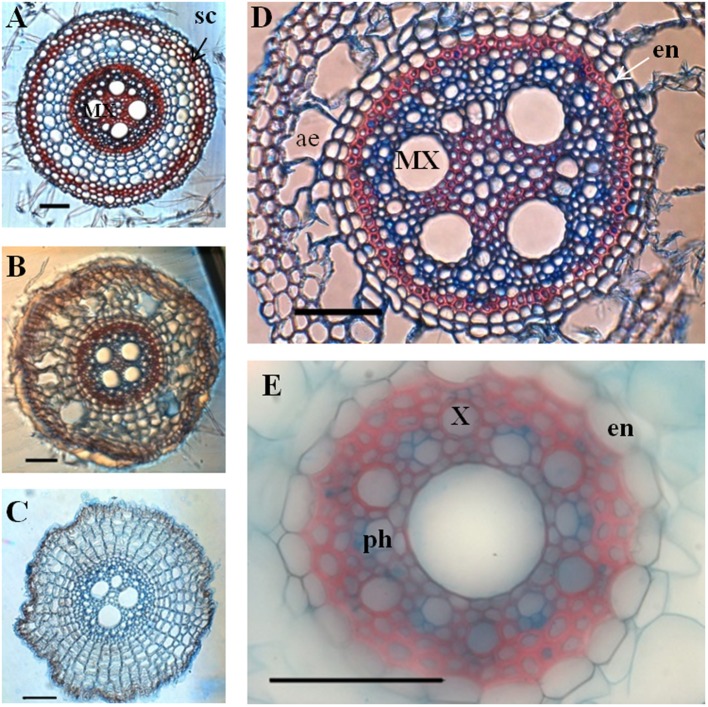
**Transverse section of crown roots and primary root stained with FASGA.** Sections were performed at various level along the roots axis. **(A–C)** Transverse section of crown root, after FASGA staining. **(D)** Transverse section of a crown root after FASGA staining, focus on the stele. **(E)** Transverse section of primary root after FASGA staining, focused on the stele (scale bar: 100 μm) sc, schlerechyma; en, endodermis; X, xylem vessel; MX, metaxylem vessel; ph, phloem vessel; ae, aerenchyma.

In order to localize secondary deposition (lignin or suberin) in the cell wall, we performed FASGA staining on transverse sections of primary and crown roots (**Figure 5**). The formation of a typical horseshoe-shaped Casparian strip could be visualized in the endodermis of both primary and crown roots as they differentiated. In addition, the FASGA staining revealed six xylem poles, alternating with six phloem poles in the primary root (**Figure 5E**), while we observed 12–16 xylem poles in crown roots (**Figure 5D**). Mature parts of crown roots displayed a sclerenchyma, surrounded by a hypodermis and a rhizodermis (**Figure 5A**).

**FIGURE 6:**
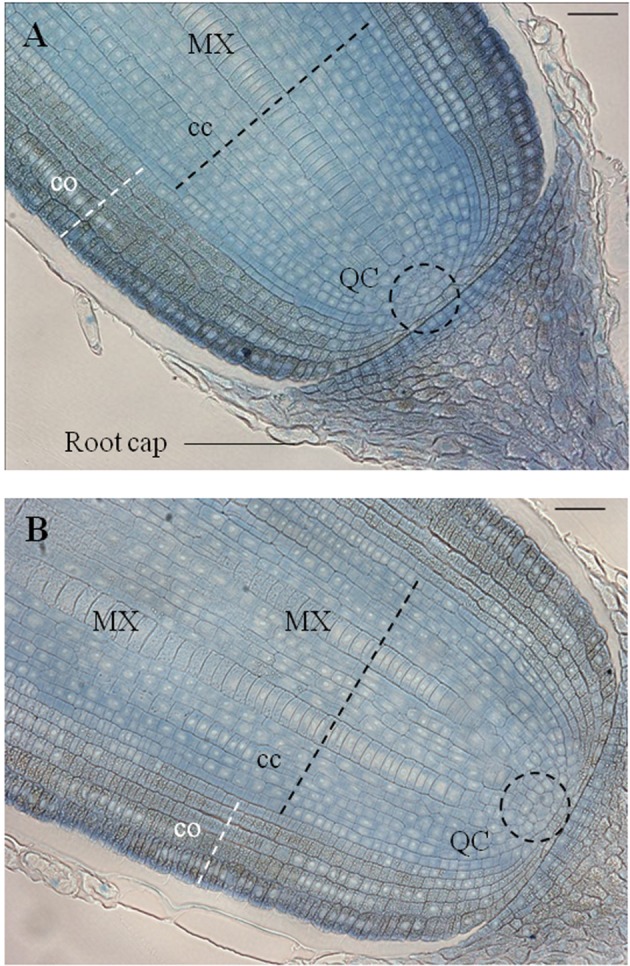
**Anatomical organization of primary root and crown apices observed on a longitudinal section, stained with toluidine blue, sampled 5 DAG. (A)** Longitudinal section of a primary root apex. **(B)** Longitudinal section of a crown root apex. QC, quiescent center; cc, central cylinder; co, cortex; MX, metaxylem vessel (scale bar: 100 μm).

Longitudinal sections (5 μm) through the primary root meristem revealed a closed meristem organization with cell files converging to a small group of cells whose location and size are consistent with those of quiescent center cells (**Figure 6A**). The metaxylem differentiated and expanded radially close to the putative initial cells. Cortex parenchyma cells accumulate metabolites, possibly starch grains, but further investigation is needed to identify the nature of this deposit. Longitudinal sections through the crown root meristem showed a similar closed meristem organization with a larger stele (**Figure 6B**).

**FIGURE 7:**
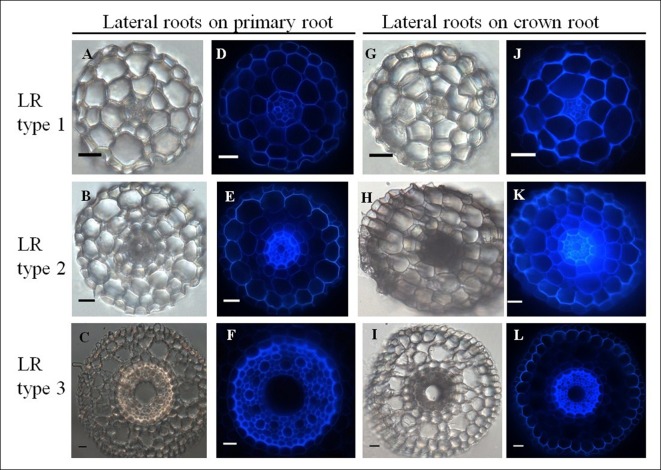
**Comparative anatomical organization of lateral roots (left: transmitted light, right: autofluorescence). (A–F)** Lateral root emerging from primary root. Picture F only shows the root stele. **(G–L)** Lateral root emerging from crown root. Three root types are identified, independent of the mother root: LR type 1: small root diameter and no metaxylem **(A,D,G,J)**, LR type 2: medium root diameter and small diameter metaxylem vessel, **(B,E,H,K)**, LR type 3: large root diameter and large diameter central metaxylem vessel: **(C,F,I,L)**. Scale bar: 20 μm.

Transverse sections through first order lateral roots (*n* = 33) branching from either primary or crown roots revealed distinct organizations. Interestingly, lateral roots could be classified into three types based on their anatomy (**Figure 7**, **Table 1**). Type 1 lateral roots are very thin (68–140 μm diameter) with an anatomy characterized by a diarch (two protoxylem poles) stele without any central metaxylem vessel. Ground tissues include an endodermis, a bi-layered cortex, and epidermis, but neither sclerenchyma nor aerenchyma (**Figures 7A,D,G,J**). Type 2 lateral roots have a medium diameter (235–291 μm), show one small (16 μm diameter in average) metaxylem vessel and three layers of cortical cells. Like type 1, type 2 lateral roots have no sclerenchyma or aerenchyma (**Figures 7B,E,H,K**). Finally, type 3 lateral root exhibit the largest diameter (328–440 μm similar to primary root) and the same organization as primary roots, independently of the root from which they emerge (i.e., primary root or crown root; **Figures 7C,F,I,L**). Hence our anatomical studies have revealed that there are three distinct types of lateral roots that form on both the primary root and crown roots in pearl millet.

### Diversity in Pearl Millet Root Development

We next addressed whether there was significant variation in pearl millet root architecture. We selected 16 lines from a panel of pearl millet inbred lines (Saïdou et al., 2009). As our objective was to maximize diversity, these lines were sampled to represent the whole diversity observed in the phylogenetic tree of 90 inbred lines (Saïdou et al., 2009), taking also into account a sufficient seed set availability and good germination rate. We analyzed the root system of these plants using a germination-paper-based phenotyping platform (Atkinson et al., 2015).

**FIGURE 8:**
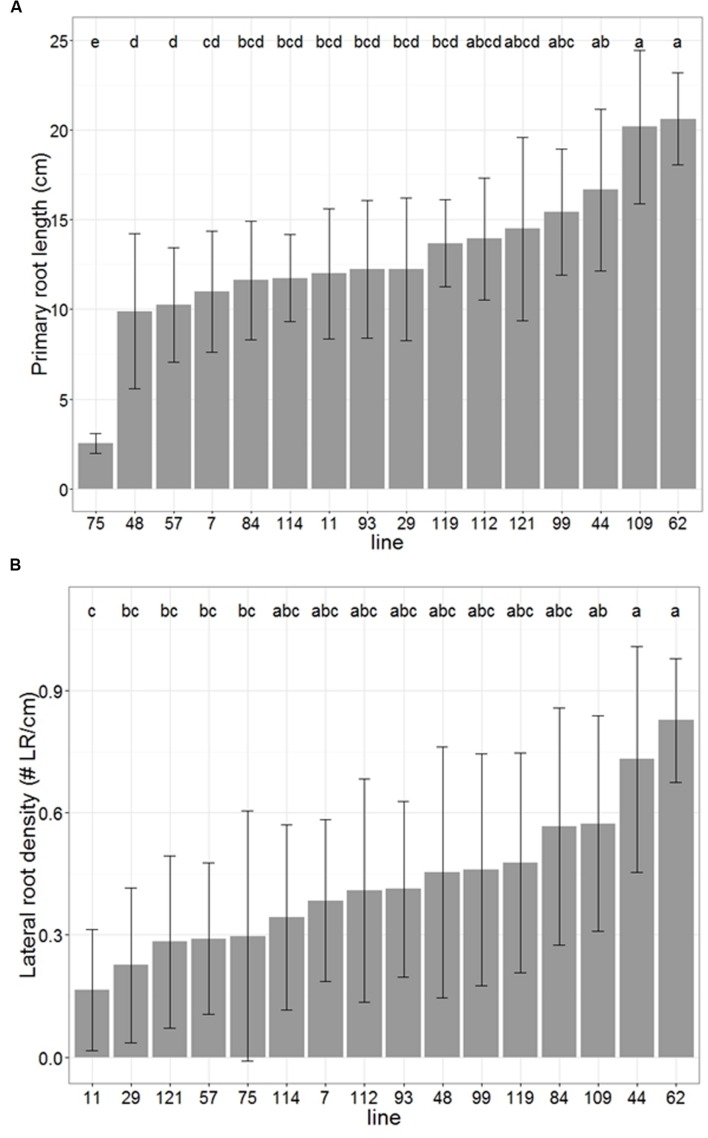
**High throughput pearl millet root phenotyping: distribution of primary root length **(A)** and lateral root density **(B)** among 16 pearl millet from a panel of inbred lines covering a large genetic diversity.** Error bars represent standard deviation, letters represent Tukey’s HSD groups.

We observed large variation in primary root growth and lateral root density along the primary root among the individuals screened of this panel (**Figure 8**). In both cases, a significant part of this variability was explained by the genetic line variable (ANOVA *p* < 0.01). The lines could be separated into groups of homogeneous means with a Tukey’s HSD test. For primary root length, the group identification showed some clear outliers with especially large or small values, associated with a group of lines with intermediate and quite homogeneous values (**Figure 8A**). For lateral root density, no clear outlier was observed, the values for all the lines forming a rather smooth continuum between small and large values (**Figure 8B**). The broad-sense heritability was equal to 0.72 for primary root length and to 0.34 for lateral root density. We tested whether the variability in early primary root growth was due to differences in available seed reserves (Supplementary Figure 2) by computing the Spearman’s rank correlation coefficient between average seed weight and primary root length for each line. The Spearman’s rank coefficient correlation was equal to 0.22. This value was not significantly different to zero (*p* = 0.21), indicating that no correlation could be found between seed weights and primary root length in our experiments. As seed mainly contains reserves, this result suggests that the differences we observed are not simply due to available reserves.

## Discussion

Here, we analyzed root system architecture at early stages of the pearl millet life cycle. We named the different roots following the current standards in terms of monocotyledonous root nomenclature (Hochholdinger et al., 2004). One striking feature of early pearl millet root development is the very rapid emergence and vertical growth of the primary root (7 cm day^-1^ in our experimental conditions) compared to other cereals (3 cm day^-1^ for maize and wheat; Pritchard et al., 1987; Pahlavanian and Silk, 1988; Muller et al., 1998). In contrast, root branching started relatively late after seedling germination (6 DAG). The X-ray CT experiment confirmed this global dynamics of early root system formation. Traditionally, pearl millet is sown at the very start of the rainy season. As it was domesticated in Sahel (Oumar et al., 2008) and is mostly grown in areas characterized by light soils with a low carbon content and water retention capacity, we hypothesize that the observed developmental pattern can be favorable to the rapid colonization of deep soil horizons that retain some water. This might therefore be an important adaptive strategy to deal with early drought stress. The observed anatomy of pearl millet roots is consistent with those found in other cereals such as rice (Rebouillat et al., 2009), wheat, barley and triticale (Watt et al., 2008), or maize (Hochholdinger and Tuberosa, 2009). A striking difference between the different root types comes from the number of central metaxylem vessels: one (or two) in the primary root, always more than two in the crown roots, including the root emerging from the scutellar and coleoptile node. Interestingly, our analyses identified three different lateral root types on the basis of their diameter and radial anatomy. Variation in lateral root anatomy has been reported in other cereals, with numbers of distinct types varying from two in rice (Rebouillat et al., 2009) to five in wheat (Watt et al., 2008). Recently, a more detailed characterization of cortex cell layers present in rice lateral roots revealed that three types of lateral roots exist in rice (Henry et al., 2016). These anatomical distinctions share similar features across species, the smallest root type having a very simple organization, with only two (or three) xylem vessels and no aerenchyma, and the bigger type having an organization similar to a primary root. One can hypothesize that these different lateral root types have different roles: type 1 lateral roots may be involved in the exploitation of resources close to the root whilst type 3 lateral root could be involved in the branching of the root system and the exploration of new soil volumes. The role of type 2 lateral roots is still unclear. Nevertheless, the functional relevance of these differences in anatomy needs to be explored. Similarly, it will be interesting to unravel how these different lateral roots develop and how their formation is controlled by environmental factors. Whilst the molecular mechanism controlling lateral root development has been extensively studied in the model plant *Arabidopsis thaliana* (see Lavenus et al., 2013, for review), how these mechanisms are modified to form different types of lateral roots in Monocots is completely unknown.

Root phenotyping of different pearl millet inbred lines revealed a high variability for two root traits within the panel, consistent with an earlier study (Brück et al., 2003). Here, we showed that this variability was also visible *in vitro* at a very early stage of growth (6 DAG). This finding together with the high heritability of the primary root length could be exploited to identify the genetic determinants of primary root growth, a potentially beneficial root trait for pearl millet early establishment. For instance, screening of natural variability of the primary root length have been done at the cellular level in *Arabidopsis thaliana* and led to the identification of a root meristem regulator gene (Meijón et al., 2014). Beside, it will be interesting to exploit the large diversity we observed for primary root growth to test the adaptive value of this character for early drought stress tolerance.

## Conclusion

Our analysis opens the way to dissecting the genetic determinants controlling key root phenes and the characterization of their impact on yield and stress tolerance in pearl millet.

## Author Contributions

SP, PG, DW, J-LV, YV, YG, BM, and LL designed the study. SP, FG, DM, ML, SG, BMO, JA, MNB, LL performed the experiments. SP, ML, SG, BMO, MJB, DW, J-LV, YG, BM, and LL analyzed the data. SP, J-LV, YG, and LL wrote the paper. All authors read and approved the manuscript.

## Conflict of Interest Statement

The authors declare that the research was conducted in the absence of any commercial or financial relationships that could be construed as a potential conflict of interest.
