# Supplementary material for: Characterization of Pearl Millet Root Architecture and Anatomy Reveals Three Types of Lateral Roots
Source: Front Plant Sci. 2016 Jun 13;7:829. doi: 10.3389/fpls.2016.00829 (PMC4904005; doi:10.3389/fpls.2016.00829)
Supplement: Supplementary file 1 [file Image_1.PDF]

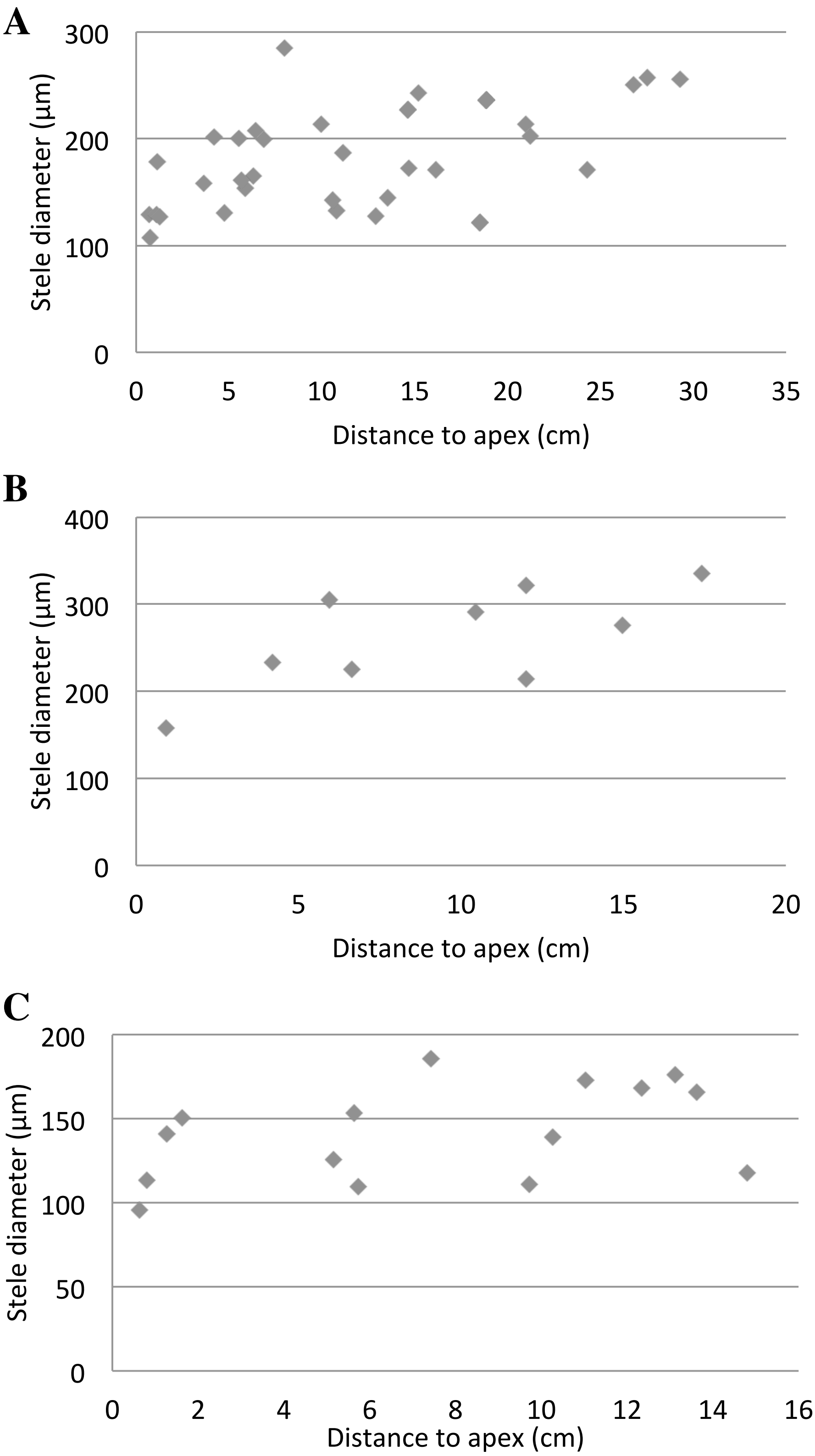

Supplementary Figure 1: Stele diameter of primary root (A) crown root (B) and type 3 lateral root (C) measured at different distances to the root apex.
