# Supplementary material for: Characterization of Pearl Millet Root Architecture and Anatomy Reveals Three Types of Lateral Roots
Source: Front Plant Sci. 2016 Jun 13;7:829. doi: 10.3389/fpls.2016.00829 (PMC4904005; doi:10.3389/fpls.2016.00829)
Supplement: Supplementary file 2 [file Image_2.PDF]

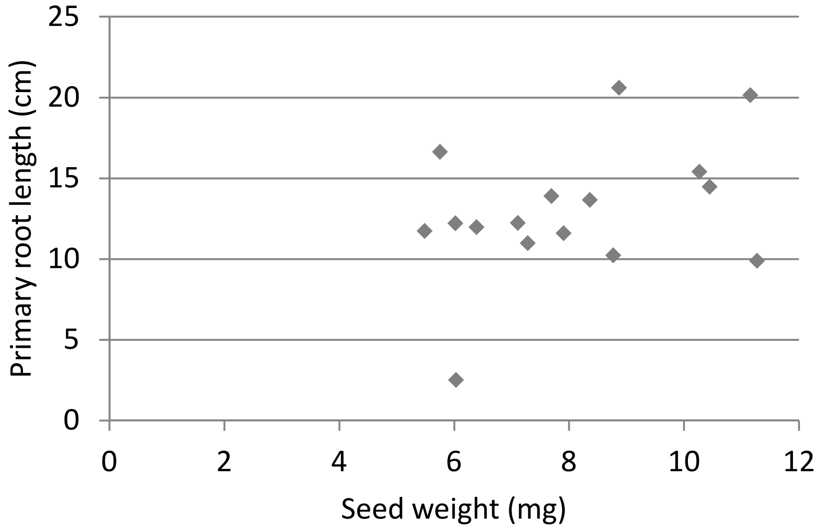

Supplementary Figure 2 : Seed weight of each line against average primary root length after 6 DAG, measured in pouches.
